# Dietary Alpha-Ketoglutarate Supplementation Improves Bone Growth, Phosphorus Digestion, and Growth Performance in Piglets

**DOI:** 10.3390/ani13040569

**Published:** 2023-02-06

**Authors:** Junquan Tian, Fan Yang, Xuetai Bao, Qian Jiang, Yuying Li, Kang Yao, Yulong Yin

**Affiliations:** 1 National Engineering Laboratory for Pollution Control and Waste Utilization in Livestock and Poultry Production, Laboratory of Animal Nutritional Physiology and Metabolic Process, Key Laboratory of Agro-Ecological Processes in Subtropical Region, Institute of Subtropical Agriculture, Chinese Academy of Sciences, Changsha 410125, China; 2 Institute of Subtropical Agriculture, Chinese Academy of Sciences, University of Chinese Academy of Sciences, Beijing 100008, China; 3 College of Animal Science and Technology, Hunan Agricultural University, Changsha 410000, China; 4 Institute of Bast Fiber Crops, Chinese Academy of Agricultural Sciences, Changsha 410205, China

**Keywords:** alpha-ketoglutarate, bone growth, apparent digestibility of phosphorus, piglets

## Abstract

**Simple Summary:**

Intensive farming in the modern pig industry has resulted in an increase in phosphorus pollution, which is a major environmental concern. The dietary intake of phosphorus is primarily employed for the growth and metabolism of pig bones. Dietary strategies to improve bone growth can be utilized to optimize the utilization of dietary phosphorus and reduce its release into the environment. Interestingly, recent studies have shown that alpha-ketoglutarate, an intermediate metabolite in the tricarboxylic acid cycle, improves osteogenesis in vitro. Therefore, we hypothesized that dietary alpha-ketoglutarate supplementation would have a positive effect on bone growth, and thereby improve the utilization of dietary phosphorus and calcium in piglets. In the present study, we found that dietary alpha-ketoglutarate supplementation improves bone growth, such as bone density, length, and weight in piglets. Of note, our study further demonstrated that alpha-ketoglutarate supplementation improves the apparent ileal and total tract digestibility of phosphorus and calcium in piglets’ diets. Our findings may provide a nutritional strategy for diminishing phosphorus pollution originating from the pig industry.

**Abstract:**

Phosphorus (P) pollution from modern swine production is a major environmental problem. Dietary interventions to promote bone growth can improve the utilization of dietary P, and thereby reduce its emission. Recent in vitro studies have shown that alpha-ketoglutarate (AKG) exerts a pro-osteogenic effect on osteoblast cells. This study aimed to evaluate the effects of AKG supplementation on bone growth, P and Ca digestion, and the gut microbial profile in piglets. Thirty-two piglets were randomly assigned into two dietary groups. The piglets were fed a basic diet containing 10 g/kg AKG or 10 g/kg maize starch (control) for 28 days. On days 21–28, titanium dioxide was used as an indicator to determine the apparent digestibility of P. AKG supplementation improved the bone mineral density, length, weight, and geometrical and strength properties of the femur and tibia. Furthermore, AKG supplementation increased apparent ileal and total tract digestibility of P. Colonic microbiota analysis results showed that AKG supplementation increased α-diversity and beneficial bacteria, including *Lactobacillus* and *Clostridium butyricum*, and decreased nitrogen fixation and chemoheterotrophy. Together, AKG supplementation improves bone growth, the utilization of dietary P, and the colonic microbial profile, which may provide a nutritional strategy for diminishing P pollution originating from the pig industry.

## 1. Introduction

Phosphorus (P) emission is one of the main causes of environmental pollution that constrains intensive farming in modern swine production [1,2]. The vast majority of P in pigs is enriched in their bones [3]. P in the diet is mainly used for the growth and metabolism of pig bones [3]. It has been proposed that dietary intervention to enhance bone growth is an effective way to improve the utilization of dietary P and reduce its emission into the environment [4,5,6].

Alpha-ketoglutarate (AKG) is an intermediate metabolite in the tricarboxylic acid cycle and transamination of amino acids, which is generated from isocitrate via isocitrate dehydrogenase and amino acids metabolism [7]. In addition, intracellular AKG is the obligate co-substrate of AKG-dependent dioxygenases, such as the ten-eleven translocation family of 5-methylcytosine hydroxylases, histone demethylases, prolyl hydroxylases, and collagen prolyl-4-hydroxylase, whereas extracellular AKG is the specific ligand of cell membrane receptor GPR99 (also known as OXGR1) [7,8,9]. Accumulated evidence suggests that AKG plays a crucial role in regulating a wide range of cellular biological processes [7,10,11]. Of note, recent studies have revealed that AKG exerts a pro-osteogenic effect on osteoblast cells in vitro [12,13]. The maintenance of bone homeostasis is dependent upon the equilibrium between osteoblast-mediated bone matrix formation and bone osteoclast-mediated resorption in adults, whereas in adolescence, bone homeostasis tends to be dominated by osteoblast-mediated bone matrix formation to support bone growth and development [14,15]. Moreover, the application of dietary nutritional interventions to promote osteogenesis has been proven to be an effective strategy to improve bone growth in young animals [16,17]. Therefore, we hypothesized that dietary AKG supplementation would enhance bone growth and development, thereby optimizing the utilization of dietary P and Ca in piglets. Previous studies have demonstrated that daily administration of AKG effectively improves the morphometry, geometrical and mechanical properties, cortical bone density, and collagen synthesis of bone in piglets [18,19]. Of note, our recent study revealed that dietary 10 g/kg AKG supplementation enhances the total tract digestibility of both Ca and P in growing pigs [20]. The bones of piglets progress quickly in terms of growth and development. Enhancing the utilization of P during the piglet stage can be a viable solution to reduce P pollution originating from the pig industry. However, the effect of dietary AKG supplementation on the utilization of dietary Ca and P in piglets is still unknown. 

Emerging knowledge suggests an increasing importance of gut microbiota in the nutrient absorption, immunity, and growth performance of piglets [21,22]. Accumulated evidence suggests that gut microbiota plays a critical role in skeletal homeostasis, as it affects the metabolic process, immune system, and hormonal secretion of the host [23,24]. Furthermore, short-chain fatty acids (SCFAs), metabolites of colonic microbiota, have been proven to increase bone mass and prevent inflammation and menopause-induced bone loss by inhibiting osteoclast differentiation and bone resorption in vivo and in vitro [25]. Our previous studies showed that dietary AKG supplementation altered the bacteria profiles in the feces of sows, and improved the colonic microbiota profiles of chronic oxidative stress model piglets [26,27]. However, the effects of AKG supplementation on colonic microbiota profiles and SCFAs, as well as the relationship between colonic microbiota and bone growth in piglets, remain largely unknown.

In this study, we evaluated the effect of dietary AKG supplementation on the bone mineral density (BMD), morphometry, the geometrical and mechanical properties of femur and tibia, the apparent ileal and total tract digestibility of P and Ca, and the colonic microbiota profiles in piglets.

## 2. Materials and Methods

### 2.1. Animal Care and Experimental Design

**Figure 1:**
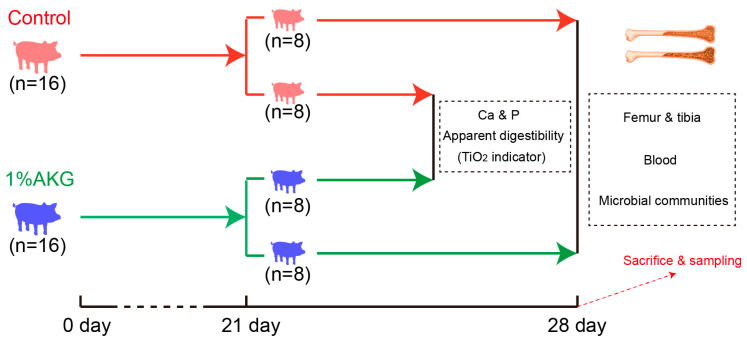
Scheme showing the experiment design.

The experiment design and procedures used in this study were approved by the Animal Ethical Committee of the Institute of Subtropical Agriculture, Chinese Academy of Science (Permit No. ISA 2020045). A total of 32 Duroc × Landrace × Large Yorkshire crossbred piglets with the same paternal origin were randomly divided into two groups (n = 16/group) at day 7 after weaning (30 days of age). Piglets in the AKG group were fed a basal diet containing 1% AKG (Wuhan Yuancheng Gongchuang Technology Co., Ltd., Wuhan, Hubei, China; purity ≥98%), the dosage of which was selected according to our previous studies [20,28,29]. To balance the ratio of carbon to nitrogen in the diet, piglets in the control group were fed a basal diet containing 1% maize starch. During a 28-day study, each piglet was kept in its own pen with a hard plastic slatted flooring and had ad libitum access to the experimental diets and water. Feed intake and weight gain were monitored during the experiment (Figure 1). The basal diet was formulated in accordance with the recommended nutrient requirements of the National Research Council (2012). The components of the basal diet are provided in Table 1. 

On the 21th day of the experiment, 8 piglets were chosen randomly from each group to perform the Ca and P digestibility test. Titanium dioxide (TiO_2_) was uniformly included in all experimental diets in a 0.5% proportion to act as an indicator. After administering the diet with the indicator for 3 days, approximately 50 g of feces were collected and stored at −20 °C from each pig every morning for 3 consecutive days. On day 28, four hours after the piglets had consumed the experimental diet, they were anesthetized with sodium pentobarbital intravenously (50 mg/kg BW) and bled by exsanguination. Approximately 50 g digesta from the terminal ileum was collected and stored at −20 °C.

On the 28th day of the experiment, after overnight fasting, blood samples were taken from the anterior vena cava of the remaining 8 piglets in each group; then, piglets were anesthetized with sodium pentobarbital intravenously (50 mg/kg BW) and bled by exsanguination. The femur, tibia, and metatarsal were collected and stored at −20 °C. The colonic digesta were collected, immediately frozen in liquid nitrogen, and stored at −80 °C. The scheme of the experiment design is shown in Figure 1.

### 2.2. Apparent Ileal Digestibility of Ca and P Analysis

The samples of fecal, ileal digesta, and diet were dried at 65 °C in an oven and then passed through a 0.5-mm screen. The dry matter of the samples was measured after oven-drying at 105 °C for 24 h. The Ca, P, and TiO_2_ contents of the samples were ascertained using the method described in the preceding literature [30]. Apparent ileal digestibility of Ca and P and apparent total tract digestibility of Ca and P were calculated using the following equations:

(1) $$\mathrm{Apparent}\;\mathrm{ileal}\;\mathrm{digestibility}\;\mathrm{of}\;\mathrm{Ca}=\left[1-\left(\frac{{\mathrm{TiO}}_{2\;\mathrm{diet}}}{{\mathrm{TiO}}_{2\;\mathrm{ileal}\;\mathrm{digestia}}}\right)\times \left(\frac{{\mathrm{Ca}\;}_{\mathrm{ileal}\;\mathrm{digestia}}}{{\;\mathrm{Ca}}_{\mathrm{diet}}}\right)\right]$$

(2) $$\mathrm{Apparent}\;\mathrm{ileal}\;\mathrm{digestibility}\;\mathrm{of}\;P=\left[1-\left(\frac{{\mathrm{TiO}}_{2\;\mathrm{diet}}}{{\mathrm{TiO}}_{2\;\mathrm{ileal}\;\mathrm{digestia}}}\right)\times \left(\frac{{P\;}_{\mathrm{ileal}\;\mathrm{digestia}}}{{\;P}_{\mathrm{diet}}}\right)\right]$$

(3) $$\mathrm{Apparent}\;\mathrm{total}\;\mathrm{tract}\;\mathrm{digestibility}\;\mathrm{of}\;\mathrm{Ca}\;=\left[1-\left(\frac{{\mathrm{TiO}}_{2\;\mathrm{diet}}}{{\mathrm{TiO}}_{2\;\mathrm{fecal}}}\right)\times \left(\frac{{\mathrm{Ca}\;}_{\mathrm{fecal}}}{{\;\mathrm{Ca}}_{\mathrm{diet}}}\right)\right]$$

(4) $$\mathrm{Apparent}\;\mathrm{total}\;\mathrm{tract}\;\mathrm{digestibility}\;\mathrm{of}\;P\;=\left[1-\left(\frac{{\mathrm{TiO}}_{2\;\mathrm{diet}}}{{\mathrm{TiO}}_{2\;\mathrm{fecal}}}\right)\times \left(\frac{{P\;}_{\mathrm{fecal}}}{{\;P}_{\mathrm{diet}}}\right)\right]$$

in which TiO_2 diet_, TiO_2 ileal digestia_, and TiO_2 fecal_ represent the concentration of TiO_2_ in diet, ileal digestia, and fecal, respectively; Ca _ileal digestia_, Ca _diet_, and Ca _fecal_ represent the concentration of Ca in ileal digestia, diet, and fecal, respectively; and P _ileal digestia_, P _diet_; and P _fecal_ represent the concentration of P in ileal digestia, diet, and fecal, respectively.

### 2.3. Bone Analysis

Bone analysis was conducted according to previous studies [16,17]. Briefly, the femur and tibia samples were prepared by manually discarding the surrounding skin, muscle, and other tissues, and then stored at −20 °C. In the subsequent stages of analysis, the left femur and tibia were selected to measure their midshaft geometry and histomorphometric, whereas the right femur and tibia were used to determine bone density, length, weight, and mechanical testing. In preparation for all analyses, the frozen bones were thawed for 8 h at room temperature. The whole bone mineral density (BMD) of the femur and tibia was determined using the dual-energy X-Ray Bone Densitometer (MEDILINK, Pérols, France).

The mechanical properties of the femur and tibia were determined using a Universal Testing Machine (Zwick Z010, Zwick Roell Group, Ulm, Germany) at room temperature by subjecting each bone to the three-point bending test according to previous studies [17,31]. Briefly, a diamond bandsaw was used to create a cross-section at the midpoint of the left femur and tibia. The internal and external cross-sectional diameters were determined using a digital caliper. The bone geometrical properties, including the mean relative wall thickness, cross-sectional area, cross-sectional moment of inertia, and cortical index, were calculated on the basis of bone cross-sectional diameters according to previous studies [16,32,33]. The midpoint of the bone diaphysis was subjected to a loading rate of 10 mm/min until the bone fractured. The bone-breaking strength and maximum elastic strength were determined from the load-displacement curves.

### 2.4. Bone Mineral Concentration Analyses

The levels of individual elements in the dried metatarsal were determined using an inductively coupled argon plasma spectrophotometer (ICP-OES -5110) according to previous studies [17]. In brief, the metatarsal samples were dried for 8 h at 105 °C in order to measure dry weight, and then ashed in a muffle furnace at 550 °C for 4 h. The ash was dissolved in 5% HNO_3_ before analysis. 

### 2.5. Analysis of the Indexes Related to Bone Metabolism

Blood samples were collected in aseptic-capped tubes without anticoagulant from the anterior vena cava. The blood samples were allowed to stand at room temperature to enable the blood to clot and were centrifuged at 3000× *g* for 10 min. The serum samples were collected and stored at −25 °C. Serum alkaline phosphatase (ALP), Ca, and P were determined using an automatic biochemistry analyzer (Cobas c311). Osteocalcin (H152), C-terminal cross-linking telopeptide of type I collagen (β-CTX-I) (H287), and N-terminal propeptide of type I procollagen (PINP) (H285) were measured using commercial kits according to the manufacturer’s instructions (Jiancheng Bioengineering Institute, Nanjing, China).

### 2.6. Serum-Free Amino Acid Analysis

Serum-free amino acids were analyzed by an automatic amino acid analyzer (L-8900, Hitachi Global Inc., Tokyo, Japan).

### 2.7. Gut Microbiota Analysis

Microbial genomic DNA from colonic digesta was extracted and amplified using specific primers with barcodes (16S V3 + V4). Amplicon libraries were sequenced on the Illumina MiSeq 2500 platform (Illumina, San Diego, CA, USA) for paired-end reads. Clustering of the highest quality sequences with a 97% match resulted in the production of operational taxonomic units (OTUs) by using Uparse V.7.0 in QIIME V.1.8 (http://qiime.org/, accessed on 23 December 2020). The 16S rRNA Silva database was employed to assign taxonomy to OTUs, based on the ribosomal database project (RDP). OUT-based functional predictions were conducted using FAPROTAX. The correlation between femur and tibia bone density and the colonic microbial profile was conducted using Spearman’s correlation analysis. Visualizations were constructed using R (Version 2.15.3).

### 2.8. Analysis of Short-Chain Fatty Acids (SCFAs)

The analysis of SCFA was conducted according to our previous study [34]. In brief, the colonic digesta were homogenized, after which about 1 g of each sample was taken. The samples were diluted with 5 mL distilled water, homogenized, and centrifuged at 12,000× *g* for 10 min. The supernatant was collected. The concentration of SCFA in the samples was quantified using a gas chromatograph (Agilent) with an HP-INNOWAX column A.

### 2.9. Statistical Analyses

Data are presented as the mean ± standard error of the mean (SEM). Statistical comparisons were analyzed by independent T-test. *p* < 0.05 was considered significant. 0.05 < *p* < 0.10 was considered a significant trend. SPSS software version 20.0 (SPSS Inc., Chicago, IL, USA) was used for data analysis. 

## 3. Results

### 3.1. Growth Performance

To evaluate the effect of AKG supplementation on the growth performance of piglets, both feed intake and weight gain were monitored for 0–21 days. The growth performance data are shown in Table 2. Compared with the control group, dietary 1% AKG supplementation significantly increased the average daily feed intake (ADFI) (*p* < 0.05) and average daily gain (ADG) (*p* < 0.05). However, there was no difference in initial body weight, final body weight, and the ratio of feed to weight gain (F/G) between the control and AKG groups.

### 3.2. Bone Length, Weight, Density, Macrominerals, and Microminerals

To evaluate the effect of AKG supplementation on the bone growth of piglets, the bone density, bone weight, and bone length of the femur and tibia were determined (Table 3). Compared with the control group, piglets in the AKG group had 10% greater bone density (*p* < 0.05), 11% greater bone weight (*p* < 0.05), and 3% greater bone length (*p* = 0.109) of the femur. Moreover, the bone density, bone weight, and bone length of the piglets in the AKG group were 13% (*p* < 0.05), 8% (*p* = 0.117), and 4% (*p* < 0.05) higher than those in the control group.

To evaluate the effect of AKG supplementation on BMD of piglets, the macrominerals and microminerals of the metatarsal were determined (Table 4). Compared with the control group, 1% AKG supplementation significantly increased the Zn content in the metatarsal (*p* < 0.05). However, Ca, P, Ca/P, Na, Mg, Fe, Sr, Cr, and Mn content of the metatarsal but did not differ between the control and AKG groups (*p* > 0.05).

### 3.3. Bone Geometrical and Strength Properties

**Figure 2:**
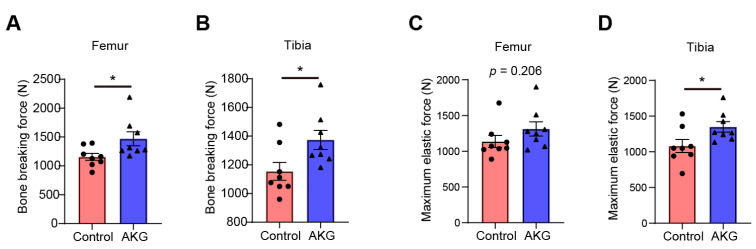
Effects of dietary supplementation with AKG on bone breaking strength and maximum elastic force of femur and tibia. (**A**) Bone breaking force of femur, (**B**) bone breaking force of femur tibia, (**C**) maximum elastic force of femur, and (**D**) maximum elastic force of tibia are shown. Data were presented as mean ± SEM (n = 8). *, *p* < 0.05.

The geometrical and strength properties of the femur and tibia were detected by a three-point bending test to evaluate the effect of AKG supplementation on the bone growth of piglets. The results are shown in Figure 2 and Table 5. Compared with the control group, 1% supplementation significantly increased the breaking force of the femur and tibia (Figure 2A,B) and the maximum elastic force of the tibia (Figure 2D) (*p* < 0.05). The maximum elastic force of the femur tended to increase in the pigs fed with AKG when compared with the control group (Figure 2C) (*p* = 0.206). A total of 1% AKG supplementation significantly increased the cross-sectional area, moment of inertia, and strain when compared with the control group (*p* < 0.05). These results suggest that dietary 1% AKG supplementation improves the geometrical and strength properties of the femur and tibia.

### 3.4. Apparent Ileal Digestibility and Apparent Total Tract Digestibility of Ca and P

To evaluate the effects of dietary AKG supplementation on the Ca and P digestion of piglets, the apparent ileal digestibility and apparent total tract digestibility of Ca and P were determined (Table 6). Compared with the control group, dietary 1% AKG supplementation significantly increased apparent ileal digestibility and apparent total tract digestibility of Ca (*p* < 0.05). The apparent ileal digestibility and apparent total tract digestibility of P in the 1% AKG group were 9.37% and 9.74% higher than those in the control group (*p* < 0.05). Next, we analyzed whether the apparent ileal digestibility and apparent total tract digestibility of Ca and P differed in the control or AKG group. As shown in Table 7, the determined apparent ileal digestibility and apparent total digestibility of Ca and P did not differ whether in the control group or in the AKG group.

### 3.5. Osteocalcin, β-CTX, PINP, ALP, Ca, P, and Free Amino Acids in Serum

The concentrations of serum indicators related to bone metabolism including osteocalcin, β-CTX, PINP, ALP, Ca, and P are determined (Table 8). Compared with the control group, dietary 1% AKG supplementation significantly increased osteocalcin (*p* < 0.05). There is no difference in β-CTX, PINP, Ca, and P between the control and AKG groups. We also measured the serum concentrations of free amino acids after an overnight fast (Table 9). The results showed that compared with the control group, dietary 1% AKG supplementation significantly decreased serum ammonia (NH_3_) (*p* < 0.05). However, the concentrations of serum-free amino acids were not different between the control and AKG groups.

### 3.6. Gut Microbial Profile and SCFAs

**Figure 3:**
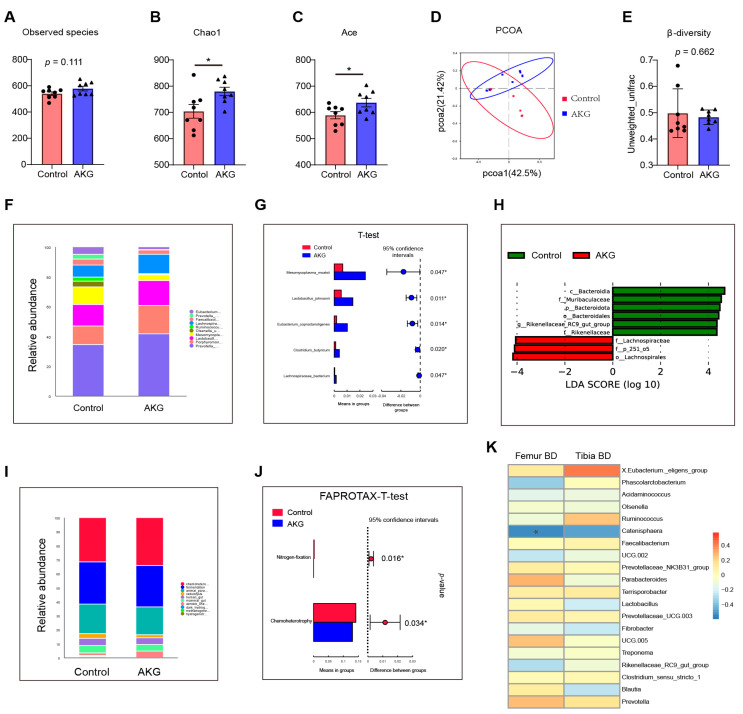
Effects of dietary supplementation with AKG on colonic microbial communities. (**A**) Observed species, (**B**) chao 1, (**C**) ace, (**D**,**E**) based on binary jaccard and unweighted unifrac, (**F**) colonic species composition, (**G**) T-test analysis of colonic species composition between control and AKG groups, (**H**) LEfSe analysis of colonic microbiota, (**I**) relative abundance of colonic microbial function, (**J**) T-test analysis of microbial function between control and AKG groups, (**K**) correlation between colonic microbial composition and femur bone mineral density (BD) and tibia bone density analysed by Spearman correlation analysis. Data were presented as mean ± SEM (n = 8). *, *p* < 0.05.

The colonic microbial profile was determined in Figure 3. The results of α-diversity showed that AKG supplementation significantly increased chao1 and ace (*p* < 0.05) (Figure 3B,C), and tended to increase in observed species (*p* = 0.111) (Figure 3 A) when compared with the control group. However, the β-diversity was not different between the control and AKG groups (Figure 3D,E). The top 10 species of relative abundance of colonic bacteria at the species level in both the control and AKG groups are shown in Figure 3F. T-tests were performed to identify the bacteria that were significantly different between the control and AKG groups at the species level (Figure 3F). The results showed that 1% AKG supplementation significantly increased *Lactobacillus johnsonii, Clostridium butyricum*, *Eubacterium coprostanoligenes*, *Lachnospiraceae bacterium*, and *Mesomycoplasma moatsii* when compared to the control group (Figure 3G). Linear discriminant analysis Effect Size (LEfSe) analysis results further showed that the order *Lachnospirales*, the family *Lachnospiraceae,* and the phylum 251 order 5 were significantly enriched in the AKG groups, whereas the class *Bacteroidia*, the family *Muribaculaceae*, the phylum *Bacteroidota*, the order *Bacteroidales*, the genus *Rikenellaceae RC9 gut group*, and the family *Rikenellaceae* were significantly enriched in the control (Figure 3H). 

To gain insight into the functional profiles of the colonic bacterial community, FAPROTAX analysis was used to predict the gene abundance of bacterial communities. The top 10 relative gene abundance of bacterial communities in both the control and AKG groups were shown in Figure 3I. FAPROTAX-T test results showed that AKG supplementation significantly decreased the metabolic pathway involved in nitrogen fixation and chemoheterotrophy as compared with the control group (Figure 3J). To determine the correlation between colonic microbiota and bone growth, we performed a Spearman correlation analysis between femur and tibia density and colonic microbial composition. The results showed that *Catenisphaera* was negatively correlated with femur density (*p* < 0.05). We also measured the concentrations of colonic SCFAs, which are colonic microbial metabolites (Table 10). Our results showed that AKG supplementation significantly increased total SCFAs when compared with the control group. However, the concentrations of acetate, propionate, isobutyrate, butyrate, isovalerate, and valerate were not different between the control and AKG groups.

## 4. Discussion

The environmental P pollution caused by intensive breeding in the modern pig industry has always been a problem. Bone is the largest P and Ca reservoir in pigs. Nutritional interventions to accelerate bone growth and development can improve P and Ca deposition in bone, and thereby reduce their emission to the environment [3]. Recent in vitro studies have demonstrated that AKG exerts a pro-osteogenic effect on osteoblast cells, which is responsible for bone matrix formation in vivo [12,13]. Piglets exhibit a swift progression in terms of bone growth and development. In the present study, we explored the potential beneficial effects of AKG supplementation on bone growth in piglets. Our results showed that dietary 1% AKG supplementation increased the BMD, length, and weight of the femur and tibia, which suggests that AKG supplementation is effective in accelerating bone growth and development in piglets. Consistent with this, a recent study reported that dietary AKG improves bone growth and development in intrauterine growth retarded (IUGR) piglets [17]. Our previous study found that dietary AKG supplementation improves bone density in growing pigs [20].

Bone growth and development are closely related to the growth performance of domestic animals. Research has shown that promoting bone growth and development can improve the growth performance and health status of piglets, and reduce the duration to get them to the market [35,36,37]. Our previous study has shown that dietary 1% AKG supplementation improves ADG and the ratio of feed to weight gain in weaning piglets [29]. Similarly, a recent study also demonstrated that dietary AKG supplementation improves body weight in IUGR piglets [17]. In this study, we found that dietary 1% AKG supplementation improves ADG and ADFI in piglets. Therefore, AKG’s potential to foster bone growth could provide a new avenue to improve the growth performance of piglets.

The influence of nutritional intervention on bone growth and development should ultimately be reflected in the geometrical and strength properties of bone. We determined the geometrical and strength properties of the femur and tibia by the three-point bending test, which suggests that dietary AKG improves the breaking strength and maximum elastic force of both the femur and tibia. The growth and development of bones in youth are crucial to the health of bones in adulthood. Studies have shown that increasing bone density as much as possible in youth can effectively ameliorate age-related osteoporosis, which results in a high risk of osteoporotic fracture in the aged population [38,39]. Of note, lameness and boar claw have a high-prevalence rate in aged sows and boars and shorten the service longevity of sows and boars [40]. The decrease in bone strength is one of the major contributing factors to lameness and boar claw in aged sows and boars [41]. Dietary AKG supplementation in young sows and boars may provide a new nutritional strategy for treating lameness and boar claw in aged sows and boars. In addition, this intriguing discovery encourages us to further explore the potential protective effects of dietary AKG supplementation on lameness and boar claw in aged sows and boars in the future.

Changes in bone metabolism can be observed through certain biomarkers in the blood. Osteocalcin is secreted to the extracellular environment by osteoblasts during bone turnover, and the release of osteocalcin will also increase with the increase of bone resorption by osteoclasts [42]. The level of osteocalcin in serum not only reflects the state of bone formation but also represents the activity of bone turnover [43]. In the present study, we found that AKG supplementation increased the levels of serum osteocalcin. However, AKG supplementation did not affect the serum levels of PINP (a marker of bone formation), β-CTX (a marker of bone resorption), Alp, Ca, or P. It is possible that these indicators are relatively insensitive to alterations in bone homeostasis caused by AKG supplementation. Collectively, these results suggest that dietary AKG supplementation accelerates bone turnover and metabolism.

Bone tissue is composed of numerous collagen, of which glycine, proline, and hydroxyproline are the most common amino acids in collagen [44]. Studies conducted in the past have revealed that dietary AKG supplementation can result in a heightened concentration of glycine and proline in the serum of animals after eating [45,46]. Moreover, an early study directly demonstrated that dietary AKG supplementation effectively stimulates bone collagen synthesis in young growing piglets [19]. However, the effect of AKG supplementation on the concentration of serum glycine and proline after an overnight fast in piglets is unclear. Our results showed that dietary AKG supplementation did not affect serum amino acid profiles. This finding further indicated that glycine and proline in serum increased by dietary AKG supplementation can be timely used for collagen synthesis. We also found that AKG supplementation caused a decrease in serum NH_3_ concentrations. This finding agrees with previous reports that dietary AKG supplementation improves the utilization of nitrogen in both a normal and a low-protein diet in pigs [20,47].

Although many in vivo studies have demonstrated that supplementations of AKG or its derivatives have positive effects on the bone under normal or pathological conditions, the effect of AKG supplementation on mineral concentrations of bone remains unclear [7]. To our knowledge, this is the first study to explore the effect of dietary AKG supplementation on the macrominerals and microminerals of bone. Our results showed that AKG supplementation increased the concentration of Zn in the metatarsal. However, AKG supplementation had no effect on macrominerals, including Ca, P, Na, and Mg, and microminerals, including Fe, Sr, Cr, and Mn. Zn supplementation has been reported to ameliorate osteoporosis in humans and animals by inhibiting osteoclast differentiation [48]. Our findings may provide a new mechanism by which AKG supplementation has positive effects on bone.

To explore whether dietary AKG supplementation to stimulate bone growth can improve the utilization of P and Ca in piglets’ diet, we used TiO_2_ as an indicator to determine the apparent total tract digestibility of P and Ca. The result showed that AKG supplementation increased the apparent total tract digestibility of P. In order to further determine whether AKG supplementation improved the absorption of P in the small intestine, we measured the apparent ileal digestibility of P and Ca. This result showed that the apparent ileal digestibility and apparent total digestibility of Ca and P had no significant difference whether in the control group or in the AKG group, which agrees with the widely accepted notion that the small is the major site of P absorption [49]. These results suggest that dietary AKG supplementation enhances the absorption of P and Ca in the small intestine of piglets. Of note, AKG has two carboxyl groups and can effectively reduce the pH value of feed, which may promote the digestion and absorption of P and Ca. Many studies have shown that dietary acidifier supplementation, such as citric acid and lactic acid, can improve the palatability of feed and thus increase the feed intake of piglets [50,51]. Consistent with this, we found that dietary AKG supplementation increased the ADFI in piglets for 0–3 weeks. Although AKG is a potential feed additive for piglets, considering its high price, future research still needs to determine its minimum effective dose.

Evidence is mounting that gut microbiota play a major part in the health status and growth performance of piglets [21,22]. The colon is the place with the largest microbial population in piglets. However, little is known about the effects of dietary AKG supplementation on colonic microbiota. In the present study, we found that AKG supplementation increased colonic microbiota α-diversity. An increase in α-diversity has been demonstrated to be beneficial for intestinal health [52]. We also found that AKG supplementation promoted the colonization of beneficial bacteria, including *Lactobacillus johnsonii* and *Clostridium butyricum* [53,54]. Functional analysis of the colon microbial profile showed that AKG supplementation decreased the metabolic pathway involved in nitrogen fixation and chemoheterotrophy. This result may be related to the fact that AKG promotes the utilization of N and other nutrients in the small intestine, thereby reducing the amount of nutrients that enter the colon. We further conducted a Spearman correlation analysis of femur and tibia density and found that *Catenisphaera* was negatively correlated with femur density. SCFAs, the metabolites of colonic microbiota, have been proven to be beneficial to intestinal and bone health [25,55]. In the present study, we found that AKG supplementation increased the concentrations of colonic total SCFAs. Collectively, dietary AKG supplementation improves the colonic microbial profile in piglets.

## 5. Conclusions

In summary, dietary 10 g/kg AKG supplementation improves bone growth, the apparent total tract and apparent ileal digestibility of P and Ca, colonic microbial profile, and growth performance in piglets, which highlights that AKG is a promising feed additive for diminishing P pollution originating from the pig industry.

## Figures and Tables

**Table 1:** Ingredients of the basic diet fed to piglets.

Ingredients (%)	Basal Diet	1% AKG Diet
Corn	62.16	62.16
Soybean meal	18.23	18.23
Maize starch	1.00	–
AKG	–	1.00
Wheat bran	0.30	0.30
Whey powder	5.00	5.00
Fish meal	5.00	5.00
Soybean oil	2.50	2.50
Glucose	2.50	2.50
Limestone	0.65	0.65
CaHPO4	0.39	0.39
Salt	0.40	0.40
Lys-HCl	0.54	0.54
Met	0.10	0.10
Thr	0.20	0.20
Try	0.03	0.03
Premix ^1^	1.00	1.00
Total	100	100
Calculated nutrient levels ^2^		
DE, MJ/kg	14.45	14.45
CP, %	18.46	18.46
Lys, %	1.37	1.37
Met, %	0.40	0.40
Met + Cys, %	0.74	0.74
Thr, %	0.80	0.80
Trp, %	0.23	0.23
Ca, %	0.74	0.74
Total P, %	0.53	0.53
Available P, %	0.36	0.36
Analyzed nutrient levels		
Ca, %	0.77	0.77
Total P, %	0.59	0.59

^1^ Provided the per kilogram of diet: vitamin A. 8750 IU; vitamin D, 2500 IU; vitamin E, 25 IU; vitamin K3, 2.5 mg; vitamin B1, 2.5 mg; vitamin B2, 6.25 mg; vitamin B6, 2.5 mg; vitamin B12, 25 μg; D-biotin, 0.10 mg; folic acid, 1.25 mg, nicotinamide, 30 mg; D-pantothenic acid, 25 mg; Zn, 100 mg; Fe, 100 mg; Cu, 20 mg; Mn, 20 mg; I, 0.3 mg; Se, 0.3 mg. ^2^ The data were calculated from data provided by Feed Database in China (2020).

**Table 2:** Growth performance of piglets.

Item	Diet Treatment		*p* Value
	Control	1% AKG	
Initial body weight, kg	9.87 ± 0.22	9.84 ± 0.25	0.934
Final body weight, kg	20.41 ± 0.55	21.76 ± 0.70	0.138
ADFI, g/d	839.88 ± 27.53	924.88 ± 29.48 *	0.044
ADG, g/d	501.64 ± 19.44	567.41 ± 21.88 *	0.032
F/G	1.68 ± 0.03	1.64 ± 0.03	0.297

* Statistical significance: *p* < 0.05. Data were presented as mean ± SEM (n = 16).

**Table 3:** Effects of dietary supplementation 1% AKG on BMD, bone weight, and bone length.

Item	Diet Treatment		*p* Value
	Control	1% AKG	
Femur			
Bone density, g/cm^2^	0.75 ± 0.027	0.83 ± 0.024 *	0.026
Bone weight, g	88.60 ± 2.08	98.42 ± 3.20 *	0.022
Bone length, mm	124.17 ± 1.47	127.98 ± 1.66	0.109
Tibia			
BMD, g/cm^2^	0.52 ± 0.01	0.59 ± 0.02 *	0.008
Bone weight, g	65.84 ± 1.74	71.86 ± 3.14	0.117
Bone length, mm	114.27 ±0.72	118.89 ± 1.42 *	0.015

* Statistical significance: *p* < 0.05. Data were presented as mean ± SEM (n = 8).

**Table 4:** Effects of dietary supplementation with AKG on metatarsal macrominerals and microminerals.

Item	Diet Treatment		*p* Value
	Control	1% AKG	
Macrominerals (g/kg)			
Ca	195.22 ± 4.09	196.00 ± 2.59	0.873
P	94.54 ± 1.63	96.69 ± 1.21	0.308
Ca/P	2.06 ± 0.01	2.03 ± 0.01	0.057
Na	8.85 ± 0.13	8.90 ± 0.10	0.765
Mg	3.68 ± 0.06	3.83 ± 0.05	0.095
Microminerals (mg/kg)			
Zn	300.00 ± 10.62	369.28 ± 18.62 *	0.008
Fe	148.47 ± 6.15	130.68 ± 7.81	0.095
Sr	47.92 ± 1.29	49.92 ± 1.32	0.299
Cr	2.42 ± 0.03	2.43 ± 0.03	0.968
Mn	1.97 ± 0.09	1.83 ± 0.06	0.242

* Statistical significance: *p* < 0.05. Data were presented as mean ± SEM (n = 8). Ca: calcium; P: phosphorus; Ca/P: the ratio of Ca to P; Na: sodium; Mg: magnesium; Zn: zinc; Fe: ferrum; Sr: strontium; Cr: chromium; Mn: manganese.

**Table 5:** Effects of dietary supplementation with AKG on femur geometrical and strength properties of femur and tibia.

Item		Femur				Tibia				*p* Value		
	Control		1% AKG		Control		1% AKG		Femur		Tibia	
Geometrical properties												
Cross-sectional area, mm^2^	67.96 ± 4.74		74.45 ± 4.18		45.48 ± 2.06		54.92 ± 3.64 *		0.322		0.040	
Moment of inertia, mm^4^	2052 ± 201		2433 ± 252		912 ± 82		1308 ± 114 *		0.257		0.014	
Strength properties												
Yield stress, MPa	4.10 ± 0.32		4.27 ± 0.54		6.77 ± 0.45		6.43 ± 0.34		0.793		0.557	
Strain	0.15 ± 0.01		0.16 ± 0.01		0.16 ± 0.01		0.19 ± 0.01 *		0.504		0.025	
Modulus of elasticity, MPa	28.22 ± 2.81		27.35 ± 2.94		41.85 ± 2.46		34.08 ± 3.05		0.834		0.067	
Maximum stress, MPa	4.34 ± 0.25		4.78 ± 0.63		7.52 ± 0.33		6.59 ± 0.43		0.532		0.105	

* Statistical significance: *p* < 0.05. Data were presented as mean ± SEM (n = 8). Mpa: N/mm^2^.

**Table 6:** Effects of dietary supplementation with AKG on apparent ileal digestibility and apparent total tract digestibility of Ca and P.

Item	Ca		*p* Value	P		*p* Value
	Control	1% AKG		Control	1% AKG	
Apparent ileal digestibility, %	58.59 ± 1.43	63.81 ± 1.42 *	0.022	50.36 ± 0.89	55.08 ± 1.39 *	0.012
Apparent total tract digestibility, %	60.25 ± 1.37	65.45 ± 1.21 *	0.013	51.12 ± 1.48	56.10 ± 0.85 *	0.014

* Statistical significance: *p* < 0.05. Data were presented as mean ± SEM (n = 8).

**Table 7:** Comparison of apparent ileal digestibility and apparent total tract digestibility of Ca and P.

Item	Control		*p* Value	1% AKG		*p* Value
	Apparent Ileal Digestibility, %	Apparent Total Tract Digestibility, %		Apparent Ileal Digestibility, %	Apparent Total Tract Digestibility, %	
Ca	58.59 ± 1.43	60.25 ± 1.37	0.417	63.81 ± 1.42	65.45 ± 1.21	0.395
P	50.36 ± 0.89	51.12 ± 1.48	0.658	55.08 ± 1.39	56.10 ± 0.85	0.541

Data were presented as mean ± SEM (n = 8).

**Table 8:** Effects of dietary supplementation with AKG on osteocalcin, β-CTX, PINP, ALP, Ca, and P in serum.

Item	Diet Treatment		*p* Value
	Control	1% AKG	
Osteocalcin, μg/L	33.70 ± 0.98	37.28 ± 1.13 *	0.031
β-CTX, μg/L	8.47 ± 0.40	8.28 ± 0.50	0.766
PINP, μg/L	97.26 ± 1.84	101.41 ± 7.28	0.589
ALP, U/L	179.25 ± 9.01	203.5 ± 10.26	0.097
Ca, mmol/L	2.37 ± 0.02	2.36 ± 0.02	0.656
P, mmol/L	3.39 ± 0.09	3.54 ± 0.08	0.230

* Statistical significance: *p* < 0.05. Data were presented as mean ± SEM (n = 8).

**Table 9:** Effects of dietary supplementation with AKG on free amino acid concentrations in serum.

Component (μmol/L)	Diet Treatment		*p* Value
	Control	1% AKG	
Tau	142.37 ± 8.87	164.88 ± 11.78	0.149
Asp	55.20 ± 2.34	55.89 ± 4.34	0.890
Thr	92.58 ± 10.65	100.65 ± 13.73	0.650
Ser	90.30 ± 4.88	93.79 ± 6.53	0.674
Glu	330.45 ± 15.28	327.01 ± 14.46	0.872
Sar	11.25 ± 0.71	10.82 ± 1.97	0.842
Gly	607.46 ± 46.01	600.16 ± 41.96	0.908
Ala	316.61 ± 11.42	310.95 ± 21.38	0.819
Cit	11.68 ± 0.61	13.03 ± 1.16	0.320
Val	131.42 ± 7.40	152.38 ± 9.07	0.095
Cys	10.84 ± 0.87	10.05 ± 0.95	0.546
Met	13.01 ± 0.66	13.85 ± 1.21	0.551
Ile	64.81 ± 4.55	74.47 ± 6.64	0.251
Leu	104.91 ± 4.66	118.28 ± 9.86	0.240
Tyr	36.26 ± 2.36	39.73 ± 6.21	0.610
Phe	53.41 ± 2.87	61.15 ± 2.46	0.060
NH_3_	326.18 ± 21.51	264.16 ± 12.84 *	0.027
Orn	56.59 ± 9.84	63.99 ± 11.75	0.636
Lys	172.52 ± 17.91	193.14 ± 29.17	0.556
His	35.94 ± 2.61	38.12 ± 2.07	0.522
Arg	104.10 ± 6.58	111.26 ± 10.23	0.566
Hypro	41.94 ± 3.19	46.57 ± 5.34	0.469
Pro	129.97 ± 4.58	147.06 ± 7.37	0.069

* Statistical significance: *p* < 0.05. Data were presented as mean ± SEM (n = 8).

**Table 10:** Effects of dietary supplementation with AKG on colonic short-chain fatty acids.

Component (μg/g)	Diet Treatment		*p* Value
	Control	1% AKG	
Acetate	323.33 ± 7.29	330.51 ± 11.79	0.612
Propionate	188.84 ± 11.74	217.21 ± 11.78	0.110
Isobutyrate	11.87 ± 1.27	14.61 ± 1.52	0.189
Butyrate	121.51 ± 12.59	140.54 ± 8.93	0.238
Isovalerate	30.88 ± 1.05	30.82 ± 2.51	0.982
Valerate	31.55 ± 3.75	40.27 ± 5.12	0.191
Total SCFAs	707.97 ± 14.90	773.95 ± 23.92 *	0.035

* Statistical significance: *p* < 0.05. Data were presented as mean ± SEM (n = 8).

## Data Availability

Not applicable.
